# Chemical Composition of the Essential Oils from the Roots of *Erigeron acris* L. and *Erigeron annuus* (L.) Pers

**DOI:** 10.3390/molecules14072458

**Published:** 2009-07-09

**Authors:** Jolanta Nazaruk, Danuta Kalemba

**Affiliations:** 1 Department of Pharmacognosy, Medical University of Białystok, Mickiewicza 2a Str. 15-089 Białystok, Poland; 2 Technical University of Łódź, Institute of General Food Chemistry, 4/10 Stefanowskiego Str. 90-924 Łódź, Poland; E-mail: danuta.kalemba@p.lodz.pl

**Keywords:** *Erigeron acris* L., *Erigeron annuus* L. (Pers.), essential oil composition, polyacetylenes

## Abstract

The chemical compositions of essential oils from the roots of *Erigeron acris* and *Erigeron annuus* were studied. The essential oils were obtained by hydrodistillation in 1.0% and 0.05% yield, respectively, and analyzed by GC, GC-MS. Fifty four and forty seven constituents were identified. Predominant constituents of both oils were poly-acetylene esters: (*Z,Z*)-matricaria ester (49.4% and 45.9%, respectively) and (*Z*)-lachnophyllum ester (37.2% and 27.5%, respectively), that were accompanied by their stereoisomers as well as appropriate lactones. Polyacetylenic compounds amounted to 92.1% of *E. acris* oil and 85.8% of *E. annuus* oil. Both oils contained the same monoterpene hydrocarbons, amounting to 4.2% and 5.8%, respectively, and traces of almost the same monoterpene oxygenated compounds. The dominant sesquiterpenes in *E. acris* were elemenes and tricyclic sesquiterpene hydrocarbons, while in *E. annuus β*-sesquiphellandrene and *β*-bisabolene dominated. After flash chromatography of essential oil from *E. acris*, fractions contained acetylene esters and acetylene lactones were obtained. The configuration about double bonds for these compounds has been elucidated on the basis of ^1^H- and ^13^C-NMR analysis.

## Introduction

The genus *Erigeron* L. belonging to the family Asteraceae (tribe Astereae), involves about 150 species occurring in the Northern Hemisphere zone, mainly in North America. Some of them were introduced to Europe. In Poland, ten *Erigeron* species exist, among which *E. acris* and *E. annuus* are the most common [1]. *E. acris* (syn. *E. acer*), common name blue fleabane, is a biennial or perennial plant growing to 50 cm. *E. annuus* (daisy fleabane) is annual plant and reaches a height of up to 150 cm. Both species posses erect, branched stem ended with inflorescences. They often settle the same places like roadsides and wastelands [2].

In Italian folk medicine roots of *E. acris* are used topically to heal toothache, bruises and arthritis [3]. *E. annuus* has been used in Chinese folk medicine for the treatment of indigestion, enteritis, epidemic hepatitis and haematuria [4]. Chloroform and *n*-hexane extracts from the roots of *E. annuus* showed moderate antiproliferative effectiveness against MCF7 cells [5]. The weak antioxidant activity of diethyl ether and ethyl acetate extracts from roots of *E. acris* was observed [6].

Chemical composition of the roots of both mentioned species has been barely examined. In the roots of *E. acris* the pyromeconic acid derivative 6′-caffeoylerigeroside and phenolic acids were identified [6]. Mainly, examinations have focused on the aerial parts of both plants. From aerial parts of *E. acris*, flavonoid compounds, phytosterols, sesquiterpenes, a diterpene and triterpenes, among others, were isolated [6,7,8,9]. As the constituents of the aerial part of *E. annuus* γ-pyranone derivatives [10,11], flavonoids [12], phenolic acids and their derivatives [13], sesquiterpenoids [4,14], cyclopentenone derivatives [15] have been reported.

The first report on volatile compounds in *E. acris* and *E. annuus* dates from the 1950s, when Sørensen and Stavholt isolated (*Z*)-lachnophyllum ester from the essential oil from flowers and the nonfloral parts of *E. acris* [16]. In essential oil from different parts of *E. annuus* the relative proportions between matricaria ester and lachnophyllum ester were estimated [17]. In a recent investigation of the essential oil from *E. acris* herb over 60 components were identified. Monoterpene and sesquiterpene hydrocarbons were the major constituents. Polyacetylenes were also identified, (*Z*)-lachnophyllum ester was present in an amount of 0.1% and (*E,Z*)-matricaria ester, together with *α*-muurolene, in an amount of 6.0% [18]. In the essential oil from different organs of *E. annuus* 44 compounds in total were identified, among them monoterpene and sesquiterpene hydrocarbons, two polyacetylenes (matricaria ester and lachnophyllum ester) and organic acids were recognized [19]. The essential oil from the herb of *E. annuus* at four stages of ontogenesis was investigated. More than 60 constituents were identified (monoterpenes, sesquiterpene hyrocarbons, oxygenated sesquiterpenes and polyacetylenes). In all cases germacrene D was the dominant compound [20].

So far the composition of essential oils from the roots of *E. acris* and *E. annuus* was very poorly. Studied. Previous examination of the essential oil from the roots of *E. annuus* led to the identification of only a few compounds (*β*-elemene, *β*-eudesmol, *α*-cadinol, lachnophyllum ester, matricaria ester and trace amount of phenols and acids) [19]. At present we possess more sensitive equipment and extended libraries of data, enabling more accurate analysis of volatile constituents in plants. This induced us to newly analyze the chemical composition of volatile compounds in *E. acris* and *E. annuus*, and in the present work, a comparative analysis of composition of the essential oil from roots of *E. acris* and *E. annuus* is reported.

## Results and Discussion

The essential oils from the roots of *E. acris* and *E. annuus*, obtained by hydrodistillation in yields of 1.0% and 0.05% (w/v, based on dried plant material), respectively, were studied. The identified components are presented in Table 1. Blue fleabane oil consists of about 60 and daisy fleabane oil of about 50 compounds. The main constituents of both oils were polyacetylene esters: (*Z,Z*)-matricaria ester (49.4% and 45.9%, respectively) and (*Z*)-lachnophyllum ester (37.2% and 27.5%). Three other stereoisomers of matricaria ester were present in smaller amounts. Beside esters, the appropriate lactones were also identified, with (*Z,Z*)-matricaria lactone and its unidentified isomer, as well as (*Z*)- and (*E*)-lachnophyllum lactones. Polyacetylenic compounds amounted to 92.1% of *E. acris* oil and 85.8% of *E. annuus* oil. Both oils contained the same monoterpene hydrocarbons, which amounted to 4.2% and 5.8%, respectively, and traces of almost the same monoterpene oxygenated compounds. Totally different was the composition of sesquiterpenes, both hydrocarbons and oxygenated derivatives in the studied species. In the *E. acris* oil elemenes and numerous tricyclic sesquiterpene hydrocarbons dominated while in the *E. annuus* oil *β*-sesquiphellandrene and *β*-bisabolene were the main compounds of this group. Contrary to the *E. acris*, in the sesquiterpene fraction of *E. annuus* oil oxygenated analogues were dominant.

After column chromatography (CC) separation of the essential oil from *E. acris* on a silica gel column, fractions containing the mixture of polyacetylenic esters: (*Z,Z*)-matricaria ester, (*Z*)-lachnophyllum ester, (*E,Z*)-matricaria ester and the mixture of polyacetylenic lactones: (*Z*)-lachnophyllum lactone, (*Z,Z*)-matricaria lactone and the unidentified isomer of matricaria lactone were obtained. The structures of components in the mixtures were confirmed by ^1^H- and ^13^C-NMR analysis and comparison with spectral data reported in literature [21,22,23].

The yield of essential oil from *E. acris* roots is relatively high, 20 times higher than from roots of *E. annuus*. In both examined sources polyacetylenes are the major constituents of the volatile fraction, contrary to the oils from aerial plant parts, where monoterpenes and sesquiterpenes predominate. Examinations of Miyazawa *et al.* showed that acetylenic compounds amounted to 35.9% in flowers, 28.5% in leaves, 31.6% in stalks and to 93.5% in roots of *E. annuus*. A similar case was observed in *E. philadelphicus*; roots contained 89.7% of acetylenic compounds, but flowers, leaves and stalks 70.8%, 33.8% and 64.8%, respectively [24]. Our research confirmed the high content of polyacetylenes in roots of *E. annuus*, but the certain difference is noticed in the abundance of these compounds, in previous work - 93.5% [24] and in present study - 85.8%, perhaps the origin of the oils is a reason for this difference.

Polyacetylenes, especially aliphatic compounds with conjugated triple and double bonds, are some of more important class of compounds present in volatile fraction of several Asteraceae species. The number of carbon atoms in the chain is often characteristic of tribes or genera [25]. C_10_-acetylenes with diyn-ene and ene-diyn-ene chromophore are typical for the genus *Erigeron*. Matricaria and lachnophyllum esters were relatively often identified [16,17,24,26,27], while appropriate lactones were listed but were faintly examined [26,27,28]. In most cases the configuration about double bonds has not been elucidated. In present work the majority of isomers of polyacetylenes in *E. acris* and *E. annuus* were identified.

For the reason that the acetylenes are bioactive compounds, e.g. matricaria and lachnophyllum esters and lactones possess antifungal and antimycobacterial properties [28,29,30], they can have the influence on the activity of examined sources.

**Table 1:** Chemical composition (%) of the essential oils from roots of *E. acris* and *E. annuus*.

Peak No.	Constituent	RI^a^	RI^b^	RI^c^	*E. acris*	*E. annuus*
1.	Hexanal	780		778	t	0.3
2.	Heptanal	882		905	t	t
3.	*α*-Pinene	933		936	0.1	0.2
4.	Sabinene	969		973	0.1	t
5.	*β*-Pinene	974		978	3.3	4.9
6.	2-Pentylfuran	979		981	0.1	0.2
7.	Myrcene	984		987	0.1	0.1
8.	*α*-Phellandrene	1000		1002	t	0.1
9.	*p*-Cymeme	1016		1015	t	0.1
10.	*β*-Phellandrene	1025 ^e^		1025	0.1	0.1
11.	Limonene	1025 ^ e^		1025	0.4	0.2
12.	(*E*)-*β*-Ocimene	1040		1041	0.1	t
13.	*γ*-Terpinene	1053		1051	t	t
14.	Terpinolene	1083		1082	t	0.1
15.	*trans*-Pinocarveol	1130		1126	t	t
16.	Pinocarvone	1146		1137	t	t
17.	*p*-Cymen-9-ol	1165		1157	t	t
18.	Terpinen-4-ol	1168		1164	t	0.1
19.	*α*-Terpineol	1179		1176	t	0.1
20.	Thymol methyl ether	1219		1215	t	t
21.	2,5-Dimethoxytoluene	1229		1226	-	t
22.	5-Caranol	1232			0.1	-
23.	*trans*-Pinocarvyl acetate	1285		1287	0.1	-
24.	(*E,E*)-Deca-2,4-dienal	1297		1291	-	0.1
25.	*cis*-Pinocarvyl acetate	1298		1300	t	-
26.	7*α*H-Silphiperfol-5-ene	1334		1329	0.2	-
27.	Eugenol	1338		1331	t	0.1
28.	Panaginsene	1343		1336	0.1	-
29.	Bicycloelemene	1350		1338	t	-
30.	*δ*-Elemene	1357		1340	1.1	-
31.	iso-*β*-Elemene	1371		1359	0.1	-
32.	*α*-Copaene	1384		1378	-	t
33.	Silphiperfol-6-ene	1385		1379	0.1	-
34.	Modephene	1391		1383	0.2	-
35.	*β*-Elemene	1394		1389	t	-
36.	*α*-Isocomene	1397		1389	0.1	-
37.	6-Hydroxythymol dimethyl ether	1403		1400	t	0.1
38.	*β*-Isocomene	1419		1411	0.1	-
39.	(*E*)-Lachnophyllum lactone	1423			0.1	0.3
40.	*β*-Caryophyllene	1428		1421	0.1	0.1
41.	Pacifigorgia-2,10-diene	1430		1422	0.1	-
42.	Geranylacetone	1432		1430	t	-
43.	Matricaria lactone^d^	1439	2317		0.1	0.6
44.	(*E*)-*β*-Farnesene	1449		1446	0.1	0.2
45.	(*E*)- Methyl isoeugenol	1455		1455	t	t
46.	*α*-Humulene	1461		1460	t	t
47.	(*Z*)-Lachnophyllum lactone	1469	2474		1.1	1.4
48.	(*Z*)-Lachnophyllum ester	1474	2252	2240	37.2	27.5
49.	(*E,Z*)-Matricaria ester	1494	2189	2181	2.0	2.6
50.	(*Z,Z*)-Matricaria ester	1498	2315	2313	49.4	45.9
51.	(*Z,Z*)-Matricaria lactone	1500	2586		2.0	7.2
52.	*β*-Bisabolene	1507		1501	-	0.6
53.	*γ*-Cadinene	1509		1507	t	-
54.	*β*-Sesquiphellandrene	1518 ^e^		1516	-	1.0
55.	(*E,E*)-Matricaria ester	1518 ^e^		2259	t	t
56.	*δ*-Cadinene	1520		1520	t	-
57.	(*Z,E*)-Matricaria ester	1534	2417	2405	0.2	0.3
58.	(*E*)-Nerolidol	1552		1553	-	0.4
59.	10-*epi*-Junenol	1586		1581	-	0.2
60.	(*E*)-Isoelemicine	1619		1614	-	0.4
61.	T-Cadinol	1637		1633	t	-
62.	*α*-Cadinol	1646		1643	t	-
63.	*α*-Eudesmol	1670		1663	-	0.3
64.	Eudesma-3,11-dien-8-one	1674		1666	-	0.8
65.	Bisabol-1-one	1721		1712	-	0.2
66.	Eudesma-3,7(11)-dien-8-one	1739		1745	-	0.3
	Total identified				98.9	97.1

Order of elution and percentages are given on apolar column Rtx-1ms. t – trace, <0.05%; ^a^ Retention indices on apolar column Rtx-1ms; ^b^ Retention indices on polar column HP-Innowax; ^c^ Retention indices of literature on DB-1 column according to MassFinder 3.1; for 1, 2 according to Wiley Register 8^th^ edn.; for 48-50, 55, 57 on DB-Wax column according to Bär and Schulze [23]; ^d^ Correct isomer not identified; ^e^ Percentages determined on HP-Innowax column.

## Experimental

### Plant material

The roots of *E. acris* were collected in June and the roots of *E. annuus* in July 2008 (full bloom period), in the vicinity of Białystok (Poland). Voucher specimens (EAC 01007 and EAN 01008) are deposited in the herbarium of the Department of Pharmacognosy, Medical University of Białystok.

### Isolation and analysis of essential oils

The essential oils were obtained by hydrodistillation for three hours of dried plant material using a glass Clevenger-type apparatus, according to European Pharmacopoeia 5.0 [31]. The analyses of the oils were carried out on a Trace GC Ultra apparatus (Thermo Electron Corporation) with FID and MS DSQ II detectors and FID-MS splitter (SGE). Operating conditions: apolar capillary column Rtx-1ms (Restek), 60 m x 0.25 mm i.d., film thickness 0.25 µm; temperature program, 50-300°C at 4 °C/min; SSL injector temperature 280°C; FID temperature 300°C; split ratio 1:20; carrier gas helium at a regular pressure 200 kPa.; polar capillary column HP-Innovax (Agilent J&W), 30 m x 0.25 mm, film thickness 0.25 µm; temperature program 50-245°C (30 min) at 4°C/min; SSL injector temp. 250 °C; FID temperature 260°C; carrier gas, helium; 0.5 mL/min; split ratio 1:20. Mass spectra were acquired over the mass range 30-400 Da, ionization voltage 70 eV; ion source temperature 200°C. The NMR spectra were recorded on a Bruker Avance II Plus spectrophotometer at 700 MHz (^1^H) and 176 MHz (^13^C), using CDCl_3_ as solvent and TMS as internal standard.

The essential oil (115 mg) from the roots of *E. acris* was chromatographed on a column with Kieselgel 60 (particle size 0.040-0.063 mm, Merck, Germany), starting the elution with *n*-pentane and gradually increasing the polarity by addition of diethyl ether. In fraction 1, eluted with *n*-pentane, the mixture of sesquiterpene hydrocarbons was identified. In fraction 2, eluted with *n*-pentane-Et_2_O (19:1), the mixture of polyacetylenic esters (13 mg) and in fraction 4, eluted with *n*-pentane-Et_2_O (8:2), the mixture of polyacetylenic lactones (25 mg) was obtained. All fractions were analysed by GC-MS on both polar and apolar column and fractions 2 and 4 additionally by NMR spectroscopy.

Fraction 2: 47% (*Z*)-lachnophyllum ester, 19% (*Z,Z*)-matricaria ester, 15% (*E,Z*)-matricaria ester and 6% (*Z,Z*)-matricaria lactone. The proportions of (*Z,Z*)- and (*E,Z*)-isomers of matricaria ester were different than in essential oil, what suggest that during flash chromatography some isomerisation took place. Fraction 4: 47% (*Z*)-lachnophyllum lactone, 28% (*Z,Z*)-matricaria lactone, 13% unidentified isomer of matricaria lactone.

Identification of components was based on the comparison of their MS spectra with those of a laboratory-made MS library, commercial libraries (NIST 98.1, Wiley Registry of Mass Spectral Data, 8^th^ Ed. and MassFinder 3.1) and with literature data [32,33] along with the retention indices on apolar column (Rtx-1, MassFinder 3.1) associated with a series of alkanes with linear interpolation (C_8_-C_26_). Identification of polyacetylenes was confirmed by comparison of their RI’s on polar column with literature data [23] and by ^1^H- and ^13^C-NMR analysis. A quantitative analysis (expressed as percentages of each component) was carried out by peak area normalization measurements without correction factors.
